# Influence of Using Different Databases and ‘Look Back’ Intervals to Define Comorbidity Profiles for Patients with Newly Diagnosed Hypertension: Implications for Health Services Researchers

**DOI:** 10.1371/journal.pone.0162074

**Published:** 2016-09-01

**Authors:** Guanmin Chen, Lisa Lix, Karen Tu, Brenda R. Hemmelgarn, Norm R. C. Campbell, Finlay A. McAlister, Hude Quan, Hypertension Outcome and Surveillance Team

**Affiliations:** 1 Department of Community Health Sciences, University of Calgary, Calgary, Alberta, Canada; 2 Institute of Public Health, University of Calgary, Calgary, Alberta, Canada; 3 Department of Community Health Sciences, University of Manitoba, Manitoba, Canada; 4 Department of Family and Community Medicine, University of Toronto/Institute for Clinical Evaluative Sciences (ICES), Toronto, Ontario, Canada; 5 Department of Medicine, University of Calgary, Calgary, Alberta, Canada; 6 Department of Pharmacology and Therapeutics, University of Calgary, Calgary, Alberta, Canada; 7 Division of General Internal Medicine, University of Alberta, Edmonton, Ontario, Canada; 8 Research Facilitation, Alberta Health Services, Calgary, Alberta, Canada; Mayo Clinic, UNITED STATES

## Abstract

**Objective:**

To determine the data sources and ‘look back’ intervals to define comorbidities.

**Data Sources:**

Hospital discharge abstracts database (DAD), physician claims, population registry and death registry from April 1, 1994 to March 31, 2010 in Alberta, Canada.

**Study Design:**

Newly-diagnosed hypertension cases from 1997 to 2008 fiscal years were identified and followed up to 12 years. We defined comorbidities using data sources and duration of retrospective observation (6 months, 1 year, 2 years, and 3 years). The C-statistics for logistic regression and concordance index (CI) for Cox model of mortality and cardiovascular disease hospitalization were used to evaluate discrimination performance for each approach of defining comorbidities.

**Principal Findings:**

The comorbidities prevalence became higher with a longer duration. Using DAD alone underestimated the prevalence by about 75%, compared to using both DAD and physician claims. The C-statistic and CI were highest when both DAD and physician claims were used, and model performance improved when observation duration increased from 6 months to one year or longer.

**Conclusion:**

The comorbidities prevalence is greatly impacted by the data source and duration of retrospective observation. A combination of DAD and physicians claims with at least one year observation duration improves predictions for cardiovascular disease and one-year mortality outcome model performance.

## Introduction

Rigorous outcome research is required to adjust for comorbidities, as failing to adjust for comorbidities may raise questions for results and lead to erroneous conclusions[1]. To measure comorbidities, previous studies have used various data sources, including hospitalization discharge abstract database (DAD)[2–7], physician claims[8–13], and drug dispensations database[14,15] and different durations of retrospective observation. DAD is often used to measure Charlson comorbidities and evaluate their association with mortality, length of stay, and health care costs [16–19]. Data from DAD, however, only records comorbidities for hospitalized patients, which is problematic since many patients with chronic conditions are managed at outpatient settings. As such, comorbidities that are defined using only one database are likely to be underestimated.

Researchers have tried various approaches to accurately defining comorbidities accurately. Wang et al. [12] developed strategies for defining comorbidities by using Medicare and Medicaid claims data. Researchers in the United States[20] and Australia [21] explored the length of “look back” required for defining comorbidities and their associations with clinical outcomes, as previous studies have indicated that the prevalence of comorbidities varies depending on the data source and length of observation period used, which impact adjusted clinical outcomes.

In our study of the occurrence, management, and outcomes related to hypertension, we have found that the majority of patients with hypertension are identified through physician claims data, while patients with severe hypertension are mainly identified from DAD data [22]. Considering the long incubation period from hypertension diagnosis to the manifestation of poor clinical outcomes, along with the number of patients who are managed in outpatient settings, we aimed to maximize the length of follow-up for outcomes and minimize the duration of observation for defining comorbidities by fully using available health information. Unfortunately, to the best of our knowledge, no existing studies have compared different data sources and durations for estimating the burden of comorbidities, and the impact these approaches have on the model performance of risk adjusted outcomes among patients with hypertension. Therefore, we conducted this study to define Charlson comorbidities using DAD and physician claims data for four durations of retrospective observation (i.e., 6 months, 1 year, 2 years, and 3 years) to explore the impact of these different approaches on mortality and cardiovascular disease outcomes, among patients with newly diagnosed hypertension.

## Study Population and Method

### Data Sources

We linked data from DAD, physician claims, population registry, and death registry in the province of Alberta, Canada from April 1, 1994 to March 31, 2010 (i.e., fiscal year) using unique personal health numbers. Data from the DAD includes all inpatients in Alberta and contains up to 16 diagnoses coded according to the International Classification of Diseases, 9^th^ revision, Clinical Modification (ICD-9-CM) prior to April 1, 2002, and up to 25 diagnoses coded according to the ICD-10 Canadian Modification (ICD-10-CA) since April 1, 2002. In Alberta, physicians submit billing claims for services to the provincial Government insurance program, regardless of their service location. When submitting these claims, at least one and up to three diagnoses, coded in ICD-9, must be provided. Physician claims data captures clinical information from patients at emergency departments, hospitals, and outpatient clinics who are covered by the Alberta provincial insurance program. Due to this universal insurance program, the program registry (also called the population registry) covers nearly all Alberta residents and contains information such as personal health number, age, sex and postal code. The death registry is updated regularly and includes an individual’s date and location of death.

### Study Population and Outcomes

We extracted patients with hypertension from our linked administrative data sources using the following ICD algorithm, which has previously been validated: “two claims within 2 years or 1 hospitalization” (sensitivity 75%, specificity 94%, positive predictive value 81%, and negative predictive value 92%) [23]. Patients with pregnancy-induced hypertension were excluded [23]. To determine newly-diagnosed (incidence) cases of hypertension, we employed a 3-year washout period so not to misclassify prevalent cases as incidence. We assigned the index date for hypertension diagnosis using the first date of physician visit or hospitalization with a hypertension diagnosis code. To ensure at least a one year follow up period for the outcomes among patients with hypertension, we included incidence cases for the fiscal years 1997 to 2008, resulting in up to a 12 year follow-up period for the study population. We excluded patients with hypertension who were not residents of Alberta or who were less than 20 years of age at the time of diagnosis.

Outcomes included all-cause mortality, determined from death registry data, and cardiovascular disease (CVD), and defined as either myocardial infarction, heart failure, or stroke. We linked the study population with data from DAD and used validated coding algorithms to define myocardial infarction (ICD-9: 410.x, 412.x; ICD-10-CA: I21.x, I22.x, I25.2), heart failure (ICD-9: 428.x; ICD-10-CA: I09.9, I11.0, I13.0, I13.2, I25.5, I42.0, I42.5-I42.9, I43.x, I50.x, P29.0), and stroke (ICD-9: 362.3, 430.x, 431.x, 433.x-436.x, excluding 433.x0 and 434.x0; ICD-10-CA: H34.1, I60.x, I61.x, I63.x, I64.x, G45.x in any diagnosis field) [24–26]. Survival time was determined using the date of hypertension diagnosis and date of death/admission for cardiovascular disease. Patients were excluded if they moved out of province or reached the end of the observation period of March 31, 2010.

### Comorbidity Definitions

Charlson comorbidities were defined using validated ICD-9 and ICD-10 coding algorithms [26]. We applied these coding algorithms to our three data sources (i.e., DAD, physician claims, and both) across four retrospective periods of observation (i.e., 6 months, 1 year, 2 years, and 3 years from the date of hypertension diagnosis). Thus, we evaluated 12 approaches to defining Charlson comorbidities. We did not use Elixhauser comorbidities that contain more conditions and are better predictors of long-term mortality than Charlson comorbidities.[27] The reason is that majority of hypertension patients are captured from physician claims databases. Diagnosis code in this database is coded using ICD-9, up to 4 digits. Defining Elixhauser comorbidities requires ICD-9-CM diagnosis codes, up to 5 digits (more precise coding system than ICD-9).0

### Statistical Methods

The prevalence of Charlson comorbidities was calculated for each of our 12 approaches. We also employed logistic regression models for one year all-cause mortality and CVD hospitalization for each of these 12 approaches. Age and sex-adjusted odds ratio (OR) was estimated for each comorbidity. We then used the Cox proportional hazard regression model for all-cause mortality and CVD hospitalization and estimated the hazard ratio (HR) for each comorbidity after adjusting for age and sex.

We assessed our model performance by using C-statistics for logistic regression and concordance index CI for Cox proportional hazard regression [28]. We used 10-fold cross validation method to evaluate the model performances. The C-statistics, CI and their 95% confidence intervals were estimated using bootstrap method with 500 resamples. All analyses were conducted using SAS version 9.4 (SAS Institute Inc., USA).

This study was approved by The Conjoint Health Research Ethics Board (CHREB), University of Calgary. The waiver of consent was also approved by CHREB because this study analyzed the health administrative data, and patient records/information was anonymized and de-identified in these databases prior to analysis, approved number: REB13-0051.

## Results

Of the 759,040 patients identified with hypertension between the 1994 and 2009 fiscal years, we included 456,263 patients with newly diagnosed hypertension. As shown in Table 1, 9.9% of these were identified using data only from DAD, 86.8% using data only from physician claims data, and 3.4% using both DAD and claims between 1997 and 2008. The follow-up period ranged from 0 to 12 years (mean: 5.7 years, median: 5.5 years) with a mortality rate of 2.8 per 1000 person-years.

**Table 1:** Characteristics of patients with newly diagnosed hypertension.

Group	N	%
Total	456263	100
Age (year)		
<50	145659	31.9
50–64	167929	36.8
65–74	81396	17.8
≥75	61279	13.4
Sex		
Male	230952	50.6
Female	225311	49.4
Data source		
Physician claims only	395864	86.8
Hospital discharge abstract only	45129	9.9
Physician claims and hospitalization discharge abstract	15270	3.4
Follow-up (year)		
Mean± Standard deviation		5.7±3.2
Range (median)		0–12 (5.5)
One year mortality		3.40%
Number of death per 1000 person years		2.8

The prevalence of each comorbidity was higher for when both DAD and physician claims data was used, compared to when either of these sources was used alone (Table 2). For the 1 year ‘look back’ period, the prevalence of having at least one Charlson comorbidity was almost twice as high in claims data than in DAD data, and was even higher when both DAD and claims data sources were used together (DAD: 15.5%, claims: 30.0%, and both: 32.6%). The prevalence also increased alongside an increased length of retrospective observation, although the increase from 2 to 3 years was less than the increase from 1 year to 2 years.

**Table 2:** Prevalence (%) of comorbidities among patients with newly diagnosed hypertension by data source and duration of retrospective observation.

	Hospitalization only				Claims only				Hospitalization and Claims			
	6M	1yr	2yrs	3yrs	6M	1yr	2yr	3yrs	6M	1yr	2yrs	3yrs
Myocardial infarction	3.3	3.8	4.5	5.0	3.9	4.3	4.9	5.3	5.0	5.6	6.5	7.0
Congestive heart Failure	2.9	3.2	3.5	3.8	4.1	4.8	5.7	6.2	5.2	5.9	6.8	7.3
Peripheral vascular disease	1.4	1.6	1.8	1.9	1.6	1.9	2.4	2.7	2.5	2.9	3.4	3.8
Cerebrovascular disease	2.5	2.7	3.0	3.1	3.9	4.4	5.2	5.7	4.5	5.1	5.8	6.4
Dementia	1.0	1.1	1.1	1.2	1.2	1.3	1.5	1.6	1.7	1.8	2.0	2.2
Chronic pulmonary disease	3.8	4.2	4.8	5.3	8.2	10.9	14.8	17.5	9.9	12.6	16.4	19.1
Connective tissue disease-rheumatic disease	0.5	0.6	0.7	0.7	0.9	1.2	1.6	1.8	1.2	1.5	1.9	2.2
Peptic ulcer disease	0.6	0.7	0.8	1.0	1.2	1.7	2.7	3.4	1.5	2.1	3.1	3.9
Diabetes with complication	0.3	0.4	0.6	0.7	0.8	0.9	1.0	1.0	1.0	1.2	1.3	1.5
Diabetes without complication	2.9	3.6	4.4	4.9	3.9	4.2	4.6	4.9	5.7	6.4	7.2	7.7
Paraplegia and hemiplegia	0.2	0.3	0.3	0.4	0.7	0.8	0.8	0.9	0.8	0.9	1.0	1.1
Renal disease	1.1	1.1	1.2	1.2	1.4	1.5	1.6	1.7	1.9	2.0	2.2	2.3
Mild liver disease	0.4	0.5	0.6	0.7	0.6	0.7	1.0	1.2	0.9	1.0	1.3	1.5
Moderate and sever Liver disease	0.1	0.1	0.1	0.1	0.1	0.2	0.2	0.2	0.2	0.2	0.2	0.2
Cancer	2.0	2.2	2.6	2.9	3.9	4.6	5.6	6.3	4.2	4.9	5.9	6.7
Metastatic carcinoma	0.3	0.4	0.5	0.5	0.7	0.8	0.9	0.9	0.9	1.0	1.1	1.2
AIDS/HIV	0.01	0.01	0.01	0.01	0.03	0.03	0.04	0.04	0.03	0.03	0.04	0.04
Any one of above conditions												
≥1	14.3	15.5	17.3	18.7	25.2	30.0	36.1	40.0	28.0	32.6	38.5	42.3
1~2	11.4	12.4	13.9	14.9	23.6	27.4	32	34.8	23.6	27.8	32.8	35.8
≥3	2.8	3.1	3.5	3.7	1.6	2.2	3.3	4.2	4.4	5.2	6.4	7.4

Risk-adjusted ORs and HRs for the Charlson comorbidities varied slightly across data sources and retrospective periods in models that used mortality (Table 3) and CVD hospitalization as the outcome. The approach that used both DAD and physician claims data had the highest C-statistics, followed by DAD data only, and physician claims data only (Table 4). For each data source, the C-statistics and CI improved for CVD hospitalization and one year mortality when the retrospective period was increased from 6 months to one year or more. The 3 year DAD and physician claims approach had the highest C-statistics and CI among these 12 approaches. The C-statistics and CI were lower for modeling CVD hospitalization as an outcome than for mortality (Table 4).

**Table 3:** Age and sex adjusted odds ratio (OR) and adjusted hazard ratio (HR) of mortality and cardiovascular disease CVD) hospitalization by duration of retrospective observation among patients with newly diagnosed hypertension.

	Mortality								CVD							
Chronic conditions	OR of 1 year mortality				HR of mortality				OR of 1 year mortality				HR of mortality			
	6M	1yr	2yrs	3yrs	6M	1yr	2yrs	3yrs	6M	1yr	2yrs	3yrs	6M	1yr	2yrs	3yrs
**Claims only**																
Myocardial infarction	2.17	2.04	1.86	1.77	1.34	1.31	1.28	1.28	2.09	1.95	1.98	1.84	1.68	1.53	1.52	1.48
Congestive heart failure	3.45	3.23	3.07	2.98	2.30	2.25	2.20	2.16	2.31	2.30	2.30	2.22	2.43	2.37	2.37	2.37
Peripheral vascular disease	1.59	1.53	1.47	1.43	1.40	1.40	1.38	1.36	1.76	1.65	1.64	1.62	2.23	2.22	2.14	2.15
Cerebrovascular disease	3.17	2.92	2.66	2.46	1.77	1.71	1.62	1.58	1.70	1.69	1.69	1.65	1.77	1.77	1.76	1.72
Dementia	3.78	3.72	3.64	3.55	2.80	2.77	2.73	2.68	1.24	1.19	1.20	1.21	1.47	1.42	1.42	1.43
Chronic pulmonary disease	1.99	1.79	1.62	1.50	1.65	1.56	1.45	1.38	1.47	1.43	1.41	1.35	1.89	1.81	1.74	1.71
Connective tissue disease- rheumatic disease	1.38	1.27	1.17	1.12	1.31	1.25	1.21	1.19	1.45	1.40	1.39	1.35	1.63	1.60	1.55	1.54
Peptic ulcer disease	1.40	1.34	1.23	1.15	1.17	1.17	1.13	1.07	1.25	1.25	1.26	1.25	1.38	1.34	1.28	1.24
Diabetes without complication	1.43	1.43	1.38	1.38	1.51	1.49	1.44	1.43	2.19	2.14	2.09	2.11	2.29	2.23	2.16	2.17
Diabetes with complication	1.63	1.46	1.45	1.52	1.57	1.53	1.56	1.58	3.75	3.32	3.27	3.22	3.90	3.72	3.55	3.45
Paraplegia and hemiplegia	1.59	1.59	1.61	1.55	1.43	1.47	1.51	1.47	1.31	1.34	1.30	1.50	1.27	1.27	1.27	1.22
Renal disease	4.19	3.97	3.78	3.62	2.40	2.31	2.22	2.19	1.37	1.32	1.29	1.26	2.76	2.64	2.58	2.55
Mild liver disease	4.38	3.93	3.24	2.96	2.89	2.74	2.34	2.23	1.89	1.78	1.65	1.64	2.21	2.03	1.85	1.81
Moderate and severe liver disease	4.96	4.09	3.85	3.83	2.46	2.40	2.31	2.47	1.97	2.05	2.15	1.98	3.15	3.06	3.10	2.75
Cancer	5.85	5.01	4.19	3.78	2.47	2.26	2.04	1.96	1.16	1.13	1.11	1.11	1.34	1.28	1.23	1.22
Metastatic carcinoma	8.46	7.92	7.69	7.21	3.39	3.40	3.18	3.08	1.68	1.64	1.63	1.67	1.68	1.65	1.57	1.55
AIDS/HIV	3.47	3.74	2.69	2.28	2.86	2.79	1.99	1.80	2.45	2.10	2.10	2.09	2.64	2.23	2.15	2.04
**Hospital abstract data only**																
Myocardial infarction	1.66	1.61	1.56	1.52	1.22	1.21	1.20	1.19	1.92	2.01	2.02	2.06	1.28	1.30	1.33	1.42
Congestive heart failure	3.25	3.12	2.98	2.96	2.05	2.00	1.97	1.98	2.37	2.44	2.42	2.31	1.45	1.42	1.34	1.55
Peripheral vascular disease	1.57	1.55	1.55	1.50	1.30	1.29	1.30	1.30	1.60	1.69	1.69	1.65	1.71	1.75	1.77	1.81
Cerebrovascular disease	3.01	2.88	2.72	2.64	1.60	1.57	1.54	1.53	1.71	1.70	1.70	1.68	1.40	1.43	1.47	1.51
Dementia	3.02	3.02	3.08	3.06	2.15	2.16	2.23	2.22	1.09	1.15	1.18	1.18	1.05	1.09	1.11	1.14
Chronic pulmonary disease	2.10	2.05	1.98	1.95	1.69	1.69	1.67	1.65	1.45	1.41	1.38	1.33	1.57	1.54	1.49	1.46
Connective tissue disease- rheumatic disease	2.24	2.18	2.04	1.92	1.72	1.71	1.64	1.60	1.52	1.44	1.39	1.33	1.54	1.46	1.40	1.37
Peptic ulcer disease	1.48	1.47	1.41	1.41	1.22	1.20	1.20	1.19	1.28	1.27	1.26	1.25	1.23	1.21	1.21	1.23
Diabetes without complication	1.51	1.51	1.47	1.45	1.47	1.47	1.45	1.45	2.06	2.01	1.95	1.98	1.90	1.87	1.89	1.89
Diabetes with complication	1.26	1.26	1.28	1.32	1.45	1.44	1.43	1.44	3.33	3.13	3.14	3.32	2.65	2.61	2.60	2.67
Paraplegia and hemiplegia	1.96	2.01	2.13	2.10	1.52	1.52	1.57	1.56	1.35	1.39	1.33	1.44	1.25	1.24	1.32	1.41
Renal disease	3.37	3.37	3.39	3.38	2.04	2.01	2.00	1.99	1.64	1.55	1.46	1.41	2.18	2.10	2.03	2.00
Mild liver disease	3.42	3.15	2.73	2.63	2.30	2.19	2.00	1.98	1.89	1.74	1.60	1.64	1.74	1.64	1.52	1.52
Moderate and severe liver disease	6.65	5.94	6.04	5.93	2.43	2.46	2.63	2.65	1.41	2.18	1.86	2.12	2.13	2.19	2.38	2.34
Cancer	4.14	4.03	3.57	3.36	2.04	2.02	1.91	1.86	1.15	1.10	1.09	1.08	1.15	1.13	1.11	1.11
Metastatic carcinoma	16.12	14.51	13.54	12.95	4.97	4.72	4.44	4.37	1.33	1.47	1.44	1.54	1.31	1.34	1.27	1.23
AIDS/HIV	4.25	4.45	4.00	3.83	5.40	4.64	4.34	4.29	1.72	1.50	1.62	1.95	1.03	1.24	1.77	1.92
**Claims and hospital abstraction data**																
Myocardial infarction	1.78	1.76	1.71	1.67	1.23	1.23	1.23	1.22	1.82	1.78	1.75	2.08	1.28	1.24	1.20	1.40
Congestive heart failure	3.07	2.91	2.79	2.76	2.02	1.99	1.97	1.97	2.45	2.44	2.35	2.72	2.01	2.04	2.08	2.11
Peripheral vascular disease	1.50	1.46	1.46	1.43	1.26	1.28	1.30	1.31	1.48	1.47	1.54	1.51	2.24	2.23	2.19	2.19
Cerebrovascular disease	2.55	2.39	2.26	2.14	1.51	1.48	1.45	1.42	2.02	2.03	2.00	2.17	1.23	1.23	1.19	1.28
Dementia	3.17	3.21	3.15	3.12	2.34	2.34	2.37	2.35	1.02	1.04	1.03	1.04	1.26	1.29	1.27	1.29
Chronic pulmonary disease	1.89	1.77	1.65	1.57	1.59	1.54	1.46	1.41	1.57	1.59	1.63	1.61	2.22	2.17	2.13	2.17
Connective tissue disease- rheumatic disease	1.73	1.61	1.43	1.32	1.44	1.39	1.31	1.27	1.58	1.64	1.62	1.67	2.14	2.06	2.09	2.09
Peptic ulcer disease	1.27	1.26	1.20	1.17	1.11	1.12	1.10	1.06	1.31	1.32	1.39	1.38	1.44	1.43	1.49	1.52
Diabetes without complication	1.51	1.50	1.46	1.44	1.47	1.47	1.45	1.45	2.29	2.27	2.25	2.28	2.46	2.43	2.38	2.40
Diabetes with complication	1.21	1.22	1.27	1.31	1.34	1.34	1.35	1.37	3.85	3.69	3.38	3.41	4.13	4.04	3.93	3.87
Paraplegia and hemiplegia	2.17	2.26	2.36	2.35	1.57	1.59	1.65	1.65	1.52	1.55	1.43	1.50	1.27	1.27	1.25	1.37
Renal disease	3.27	3.24	3.21	3.16	1.97	1.96	1.92	1.92	1.45	1.43	1.48	1.47	2.98	2.94	2.92	2.98
Mild liver disease	2.77	2.63	2.28	2.19	2.18	2.12	1.89	1.83	2.28	2.18	2.08	2.00	2.83	2.65	2.48	2.34
Moderate and severe liver disease	5.97	5.05	5.11	4.94	2.26	2.14	2.24	2.31	2.22	2.27	2.28	2.13	3.60	3.44	3.37	3.06
Cancer	3.19	2.87	2.49	2.32	1.85	1.76	1.63	1.59	1.13	1.13	1.11	1.10	1.52	1.47	1.39	1.34
Metastatic carcinoma	14.12	13.54	13.13	12.64	4.39	4.23	4.10	4.00	1.55	1.59	1.58	1.57	1.80	1.77	1.67	1.60
AIDS/HIV	2.83	2.97	2.18	1.95	2.82	2.60	1.77	1.67	2.35	2.55	2.75	2.96	2.29	2.58	2.69	2.91

**Table 4:** The C-statistics and concordance index (CI) with 95% confidence intervals using 10-fold cross validation for mortality and cardiovascular disease hospitalization among newly diagnosed hypertension by data sources and ‘look back’ intervals.

	C-statistics	CI
Mortality		
Claims only		
6 months	0.859(0.853–0.865)	0.635(0.630–0.641)
1 year	0.861(0.855–0.867)	0.636(0.631–0.642)
2 years	0.863(0.859–0.868)	0.641(0.635–0.646)
3 years	0.863(0.858–0.868)	0.642(0.637–0.647)
Hospital discharge abstract only		
6 months	0.881(0.876–0.886)	0.632(0.627–0.636)
1 year	0.891(0.885–0.897)	0.636(0.631–0.640)
2 years	0.890(0.885–0.896)	0.639(0.635–0.643)
3 years	0.892(0.886–0.897)	0.639(0.634–0.645)
Claims and Hospital discharge abstract		
6 months	0.892(0.887–0.897)	0.642(0.637–0.647)
1 year	0.911(0.906–0.915)	0.646(0.640–0.651)
2 years	0.912(0.906–0.917)	0.645(0.639–0.650)
3 years	0.912(0.907–0.917)	0.647(0.641–0.652)
Cardiovascular disease hospitalization		
Claims only		
6 months	0.733(0.728–0.738)	0.547(0.541–0.552)
1 year	0.735(0.730–0.739)	0.549(0.543–0.554)
2 years	0.738(0.732–0.744)	0.552(0.546–0.557)
3 years	0.739(0.732–0.745)	0.556(0.550–0.562)
Hospital discharge abstract only		
6 months	0.739(0.733–0.744)	0.544(0.538–0.550)
1 year	0.741(0.735–0.746)	0.546(0.541–0.552)
2 years	0.747(0.741–0.751)	0.552(0.547–0.557)
3 years	0.749(0.744–0.755)	0.554(0.549–0.560)
Claims and Hospital discharge abstract		
6 months	0.756(0.751–0.761)	0.580(0.574–0.586)
1 year	0.768(0.763–0.773)	0.611(0.606–0.616)
2 years	0.769(0.763–0.774)	0.612(0.607–0.618)
3 years	0.770(0.764–0.775)	0.614(0.609–0.619)

The C-statistics were estimated using a logistic regression model while adjusting for age group, sex and 17 Charlson conditions. The confidence intervals were estimated using bootstrap method.

CI was estimated using Cox's proportional regression model while adjusting for age group, sex, and 17 Charlson conditions. The confidence intervals were estimated using bootstrap method.

## Discussion

We found that the use of DAD data alone underestimated the prevalence of comorbidities, while use of both physician claims and DAD data with a 3-year retrospective observation period yielded the highest prevalence. The model performance for one year mortality and cardiovascular disease hospitalization was statistical significantly improved for the approach that used DAD and physician claims data when compared to the approach of using only one of these sources for one year or longer.

Preen et al. [21] found that less than 50% of comorbidities that were recorded in the five years preceding were captured in the hospital record index. Another study in the United States reported that the prevalence of comorbidities increased from 10% when using only inpatient data to 25% when using both inpatient and physician claims data [8]. Our study supports the findings from this literature. We found that 75% of comorbidities are missing when only DAD data is used compared to when both physician claims and DAD data is used, with a retrospective observation period of three years. The prevalence of having at least one Charlson comorbidity was 9.2% when the hospital records index in DAD data was used. This increased to 18.7% when we employed a 3-year retrospective observation period to DAD data. Using physician claims data further improved the identification of comorbidities, as the prevalence reached 43.2%. These studies clearly suggest that DAD and physician claims data with a long duration of observation should be used to capture comorbidity profile.

We found that the use of different data sources had a higher impact on risk adjustment model performance than the duration of retrospective observation period for mortality and CVD outcomes that were based on C-statistics and CI. One study in the United States reported their C-statistics remained the same between a 1 and 2 year observation period for combined inpatient and outpatient and data [20]. Using inpatient data, Preen et al.[21] in Australia reported that their C-statistics had little to no improvement from a 1-year to a 5-year observation period. In Canada, however, Lee et al. [29] found that increasing the duration of retrospective observation period increased the detection of comorbidities, but only marginally improved their predictive model performance for 30-day mortality. There are several possible explanations for this. First, inpatients with hypertension are more likely to be sicker than outpatients with hypertension. As such, patients with multiple conditions and poorer outcomes are captured in DAD data. Second, regardless of the service location, physician claims record conditions not only from outpatients but also from inpatients and emergency department visitors. There is therefore a huge overlap between conditions that are recorded in DAD and claims data. Third, hypertension as an outcome is determined by many factors, such as social-demographic and clinical characteristics. However, administrative data does not capture many important factors, such as the severity of a disease. As an index of case-mix, Charlson comorbidities that are defined using data may have reached the maximum capacity for predicting clinical outcomes, such as CVD, even where prevalence increases with duration. Regardless of how data may be enhanced through duration, however, we have no much margin to improve risk adjustment model performance. Fourth, patients with severe conditions visit physicians frequently and their comorbidities could be captures within a short duration.

We found that the ORs and HRs for comorbidities slightly decreased with duration of observation for both mortality and CVD hospitalizations. This decrease may in part be due to false positive comorbidities, which dilute the effect of comorbidities on poor outcomes. Patients with mild comorbidities, however, are less likely to visit their physicians and more likely to have a longer survival than patients with severe comorbidities.

Limitations to this study are as follows. First and foremost, we did not validate comorbidities that were identified in physician claims data. Previous studies have indicated that validation for chronic conditions varies for different conditions and data sources. As the observation period increased, more false positive chronic conditions were included due to ICD coding errors. These false positive conditions might influence the discriminatory ability for poor outcomes. Secondly, comorbidities were defined prior to hypertension diagnosis. Some comorbid conditions may have occurred after the diagnosis of hypertension and contributed to poor outcomes. We did not account for time-dependent variables. Thirdly, we followed patients with incident hypertension for up to 12 years. The HRs might have changed with a longer follow-up period. Lastly, hypertension and comorbidities in this study were identified using Canadian administrative health data from a universal health insurance program. Thus, the findings from our study may not be generalizable to countries with different healthcare systems.

In conclusion, using a combination of DAD and physician claims data substantially improved the capture of chronic comorbidities. Prevalence was significantly increased with an increase in the duration of a retrospective observation period. A combination of DAD and physician claims data with one year or longer observation duration observation duration improves predictive model performance for cardiovascular disease hospitalization and one year mortality outcomes, because many chronic conditions are managed in outpatient clinical settings.
